# A Novel Approach to Automated 3D Spalling Defects Inspection in Railway Tunnel Linings Using Laser Intensity and Depth Information

**DOI:** 10.3390/s21175725

**Published:** 2021-08-25

**Authors:** Mingliang Zhou, Wen Cheng, Hongwei Huang, Jiayao Chen

**Affiliations:** Key Laboratory of Geotechnical and Underground Engineering, Department of Geotechnical Engineering, Tongji University, Siping Road 1239, Shanghai 200092, China; zhoum@tongji.edu.cn (M.Z.); huanghw@tongji.edu.cn (H.H.); 1810181@tongji.edu.cn (J.C.)

**Keywords:** spalling defects inspection, laser intensity, depth image, tunnel lining defect, mobile laser scanning, deep convolutional neural network, 3D reconstruction

## Abstract

The detection of concrete spalling is critical for tunnel inspectors to assess structural risks and guarantee the daily operation of the railway tunnel. However, traditional spalling detection methods mostly rely on visual inspection or camera images taken manually, which are inefficient and unreliable. In this study, an integrated approach based on laser intensity and depth features is proposed for the automated detection and quantification of concrete spalling. The Railway Tunnel Spalling Defects (RTSD) database, containing intensity images and depth images of the tunnel linings, is established via mobile laser scanning (MLS), and the Spalling Intensity Depurator Network (SIDNet) model is proposed for automatic extraction of the concrete spalling features. The proposed model is trained, validated and tested on the established RSTD dataset with impressive results. Comparison with several other spalling detection models shows that the proposed model performs better in terms of various indicators such as MPA (0.985) and MIoU (0.925). The extra depth information obtained from MLS allows for the accurate evaluation of the volume of detected spalling defects, which is beyond the reach of traditional methods. In addition, a triangulation mesh method is implemented to reconstruct the 3D tunnel lining model and visualize the 3D inspection results. As a result, a 3D inspection report can be outputted automatically containing quantified spalling defect information along with relevant spatial coordinates. The proposed approach has been conducted on several railway tunnels in Yunnan province, China and the experimental results have proved its validity and feasibility.

## 1. Introduction

During the past two decades, there has been a rapid expansion of the transportation infrastructure system in China, and as a result the number of railway tunnels has greatly increased. As tunnel service time increases, the tunnel linings inevitably deteriorate and develop various structural defects, such as cracks, leakages, spalling, and deformation. Of these, concrete spalling is one of the most common defects on the surface of railway tunnel linings [1,2,3]. Concrete blocks may fall into the tunnel during train operations if spalling defects are not identified in time, which can cause serious accidents and significant economic losses [4]. In order to ensure the safe operation of railway tunnels, spalling defect inspection needs to be performed regularly to prevent concrete falling accidents. 

To date, as a common practice in the field, the spalling regions of railway tunnel linings are inspected by certified personnel walking along the tunnel and manually documenting the descriptions of the spalling defects in a report file. However, spalling information obtained in this way is highly subjective and often relies on the engineering knowledge of the inspectors. A manual inspection report cannot provide quantitative measurement of concrete spalling defects. Furthermore, to ensure the continuity of train services, the inspection time for the railway tunnel is often limited to a few hours in the middle of the night [5,6]. Hence, the manual inspection process is inefficient and it is difficult to meet the growing inspection demands. There is an urgent need for an advanced method to detect and evaluate spalling defects in tunnel linings automatically and accurately.

In the search for automated methods to improve the efficiency of spalling defects inspection, two vision-based techniques for tunnel inspection have gained popularity. Photogrammetry-based approaches use photographic equipment such as cameras to capture and extract features of the structural defects from the obtained images [7,8,9]. However, the 2D lining images obtained from the photographic equipment do not contain the depth and spatial location information of the detected defects in the tunnel. Furthermore, the quality of the obtained images is affected by the tunnel environment (e.g., illumination conditions, or the distance from the lining to the camera lens). As an alternative, mobile laser scanning (MLS) has become popular for performing automated inspection of the tunnel lining [6,10,11]. Based on light detection and ranging technologies (LIDAR), MLS can obtain stable 3D point cloud data. The spatial position and surface texture features of the tunnel lining are both available in the high precision 3D coordinates and the intensity values of point cloud data [5,12]. By implementing image processing (IP) [1] and roughness description [13] algorithms, the spalling targets can be segmented and the depth features of the detected spalling defects can be obtained. The localization and quantification information (e.g., area and depth) of spalling defects can be subsequently acquired [13,14]. However, such approaches fail to learn the semantic features of concrete spalling, and lack robustness and accuracy [3,15]. 

Deep convolutional neural network (DCNN), a significant branch of deep learning methods, has gained state-of-the-art performance in feature detection and segmentation [16,17]. By employing multiple convolution layers, DCNN models with various backbone networks (VGG [18], ZFNet [19], GoogleNet [20], ResNet [21], etc.) have been developed to extract object features in a semantic manner. Focusing on three major tasks (classification, object detection, and segmentation), various DCNN-based end to end models (DeepLab [22], FCN [23], Mask R-CNN [24], YOLO [25], etc.) have been proposed for detecting target objects to meet the needs of each specialized application. For inspection tasks during tunnel operation, scholars have conducted many studies into the automated detection of concrete spalling [3,26], lining cracks [27,28,29], water leakages [30,31,32], and rock mass evaluation [33,34,35] based on the DCNN models. Relying on the colour feature differences, the DCNN-based models show superior performance in distinguishing target objects from the context of the images.

Facing challenges such as complicated lining backgrounds, lighting conditions and shadow changes, improvements in the precision and accuracy of defects detection still need to be addressed [36]. Existing DCNN datasets involved in the detection of spalling defects are based on the RGB information, while the spalling depth information is missing [3,26]. For instance, concrete spalling on the tunnel lining surface may have similar colour/RGB information to the surrounding lining surface, and DCNN models often underperform in the target feature extraction process based on image datasets [37]. Moreover, utility pipelines and wires can also become obstacles in front of the spalling, which can make it impossible for models to identify the defects. 

In view of the current limitations of the DCNN methods and the image datasets, a state-of-the-art approach to solve such an issue is to employ depth images, where the pixel values present the distance information between the target point and the camera lens. RGB information combined with the depth value (RGB-D) can increase the capability of the DCNNs to detect and segment the target objects. As the first attempt at combining RGB-D and DCNNs, Lang et al. [38] discussed the performance improvement of salient object detection by including the depth information. In recent years, various RGB-D and DCNN based methods have been introduced into the field of object recognition [39,40,41,42,43,44]. Among them, D^3^Net [45] and UC-Net [46] are two representative state-of-the-art models that show promising performance. To the authors’ knowledge, no research has been made into the detection of concrete spalling defects using colour and depth combined information. Therefore, this paper shall explore the improvement and practicability over existing methods by using colour information together with depth features based DCNN approach.

In order to utilize the obtained inspection data, digital processes should be conducted to visualize and locate the detected defects on the tunnel lining surface. Image stitching/mosaicking is a common method that aids the inspector by providing an overall view of the tunnel linings [47,48]. Another popular approach is to construct a 3D tunnel model, which provides an intuitional visualization of the tunnel environment. Structure from motion (SfM) is a powerful technique for registering the individual images and construct a 3D tunnel model [49,50,51]. However, the reconstruction performance greatly depends on the illumination conditions and the quality of the tunnel lining images. Using the 3D point cloud data via MLS, the inspection result can be projected into 3D space and a high precision 3D surface model can be efficiently reconstructed [52]. Poisson reconstruction [53,54] and triangulation mesh [3,55] are two common methods to generate 3D tunnel models, which help inspectors contextualizing the location of defects in a 3D manner. Nevertheless, none of the previous studies can visualize the depth feature of the spalling defects from the constructed 2D/3D tunnel model, which should be improved by the subsequent studies.

**Figure 1:**
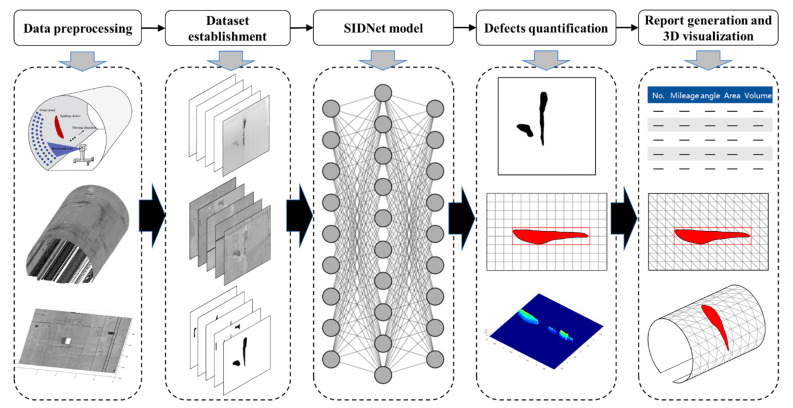
Schematic workflow of the proposed method for spalling defects inspection.

Inspired by the above-mentioned studies, in this study, an integrated approach based on the MLS, intensity and depth information, and DCNN techniques is proposed for the automated feature detection and quantification of spalling defects on the railway tunnel lining. The intensity feature obtained from the MLS system is used as the colour information during the spalling defects detection. The depth value of a spalling defect is defined as the distance from the spalling region to the lining surface. Field experimentation has been conducted, and the spalling defects inspection results demonstrate sound performance. Figure 1 shows the schematic workflow of the proposed approach, which contains five steps. An MLS system is first adopted for the acquisition of point cloud data, which obtains the spatial geometry and surface texture information of the tunnel linings. The dataset of spalling defects is then established by processing the point cloud data into 2D intensity images and depth images. To achieve automated detection of the spalling defects, a DCNN-based model named Spalling Intensity Depurator Network (SIDNet) is proposed to train the dataset. The segmentation results of the spalling defects can thus be obtained from the validated SIDNet model, and the spalling defects can be quantified by combining the segmented features with depth information. Finally, a triangulation mesh method is implemented to provide the inspectors with an intuitive 3D view of the detected spalling defects, and a 3D inspection report can also be outputted automatically containing quantified spalling defect information and its spatial coordinates. 

## 2. Data Acquisition and Dataset Establishment

### 2.1. Point Cloud Data Acquisition

**Figure 2:**
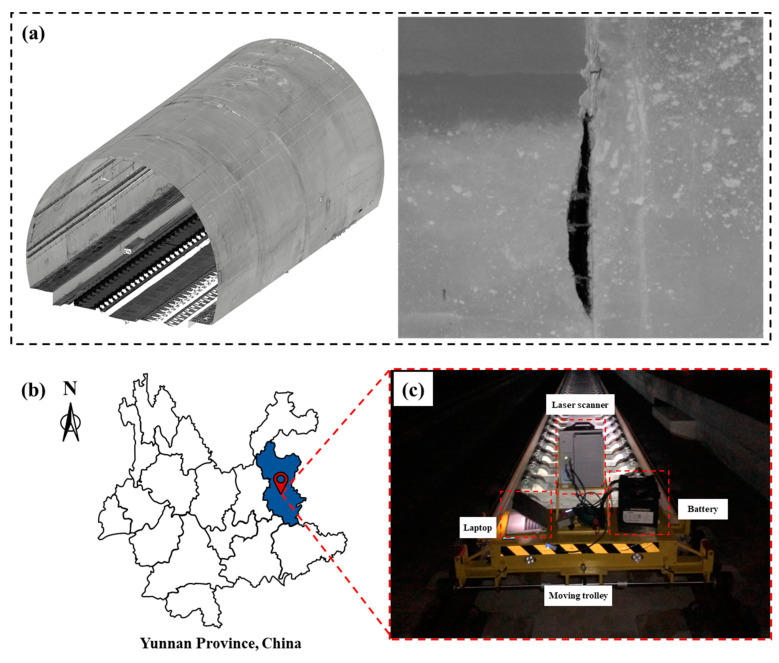
Laser scanning data acquisition: (**a**) point cloud data of the tunnel lining, (**b**) experiment location, (**c**) the MLS system.

In order to obtain both tunnel lining features (Figure 2a) and depth information of the spalling defects, an integrated MLS system [56,57] was adopted to collect the point cloud data of railway tunnel linings in Yunnan province, China (Figure 2b). The MLS system used in this study comprises a Z+F PROFILER 9012 laser scanner, an electrical motor driven trolley, a laptop, two batteries (24VDC), and other necessary accessories (Figure 2c). The PROFILER 9012 laser scanner operation is controlled by the laptop, powered by the batteries, and supported by the trolley platform. 

The tunnel is a longitudinal linear structure; therefore, the 3D tunnel point cloud data can be recorded by moving the MLS along the railway track. To ensure that the inspection process does not affect the normal operation of the railway system, the inspection time was limited from midnight to 4 a.m. The data acquisition process lasted for 14 days, and 120 Gb of MLS point cloud data were collected from 22 railway tunnels over a total length of more than 25 km.

**Figure 3:**
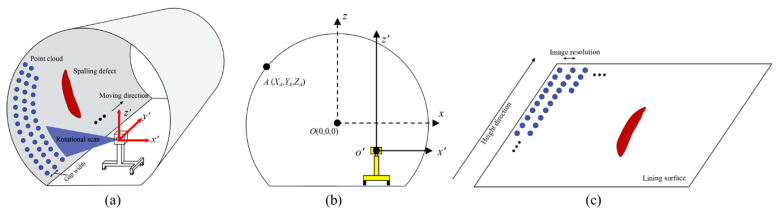
Schematic illustration of (**a**) the spalling defects scanning principle, (**b**) coordinates origin conversion to solve the unrolling distortion issue, and (**c**) 2D image generation based on the plane point cloud data.

The point cloud data collected from the MLS system include two parts: the high precision 3D coordinates and an intensity value which restores the texture of the tunnel lining. In the principle of the data acquisition, the point cloud data is collected line by line. Each scan line consists of a set of points across the transverse section of the tunnel lining. During the data acquisition, the trolley is set to move at a constant speed (0.5 m/s) and the rotation speed of the scanner is set to 100 r/s. As a result, 100 scan lines of point cloud data are recorded per second. As the rotation speed is relatively fast, the scan line of each rotation can be considered perpendicular to the Y axis. As a result, the scan lines are assumed parallel (Figure 3a) and the gap width between two adjacent scan lines can be computed as 5 mm using the equation:

(1) $$Gap=\frac{V}{F},$$

where *V* denotes the velocity of the trolley and *F* indicates the rotational frequency of the laser scanner. 

### 2.2. Intensity and Depth Images Conversion

After the point cloud data collection, the 3D high precision coordinates of each point can be obtained to represent the spatial location of the tunnel lining. The laser intensity value of each point is also collected for use in restoring the tunnel lining surface features. As a result, the point cloud data is saved as an *n* by 4-column matrix (*X*, *Y*, *Z*, *I*). In this, *X* and *Z* represent the coordinates of the tunnel transverse section, the *Y* axis represents the longitudinal direction of the tunnel (Figure 3a), and *I* represents the intensity value with a range of 0 to 255 (8 bits of data). The point cloud data can be divided into a series of lines (transverse sections) with the same *Y* coordinates.

The tunnel lining images can be generated via the image conversion process in two steps. The first step is to unroll the 3D spatial point cloud data to a 2D plane using the coordinates transformation [58]. The obtained 3D point cloud data is based on the origin at the position of the laser scanner lens, which leads to an uneven distribution of the 2D point cloud on the transverse section after the unrolling process. In order to solve this issue, the centre of the tunnel transverse section should be set prior to the unrolling process as the origin of the coordinates system [55] (Figure 3b), which can be obtained by the ellipse fitting method [59] and RANSAC algorithm [60]. The interpolation method is also used to fill in the data in the areas where the point cloud is sparse. These two approaches can effectively solve the problem of 2D point cloud distortion.

The second step is to convert the 2D plane point cloud to 2D intensity (greyscale) images. In the field of computer vision, greyscale images can be considered as a 2D matrix with each value denoting the grey value of the image ranging from 0 to 255. As a result, the 2D point cloud data with a specific number of lines can be assigned in order to a 2D matrix with the same number of columns (Figure 3c). In this regard, the longitudinal resolution of the obtained images should be the same as the distance between scan lines (5 mm/pixel). Hence, for the sake of resolution consistency, the height direction (representing the tunnel transverse section) of the obtained images shall also be set to 5 mm/pixel resolution. For each pixel in the greyscale image matrix, the intensity value in the point cloud is set as the grey value of the images. 

**Figure 4:**
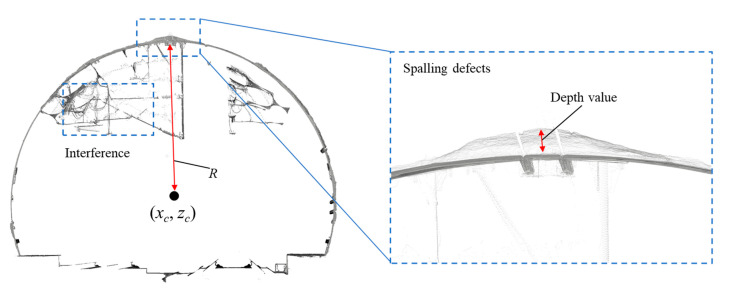
Demonstration of the spalling defects depth value from the tunnel point cloud data.

The obtained intensity images can represent the surface texture of the tunnel linings, but the depth information is missing. In order to obtain the tunnel lining depth images, the depth value of the spalling defects should be firstly computed through the following criterion:

(2) $$D=\{\begin{array}{c} \begin{array}{cc} 0 & D<0 \end{array}\\ \sqrt{{\left(x-x_{c}\right)}^{2}+{\left(z-z_{c}\right)}^{2}}-R\\ \begin{array}{cc} 1 & D>1 \end{array} \end{array},$$

where the depth value *D* denotes the absolute depth value between the spalling defects to the fitting ellipse lining surface. It is assumed that the transverse section of a deformed circular-tunnel can be considered as an ellipse [61], (*x*, *z*) is an arbitrary point from the point cloud data, (*x_c_*, *z_c_*) is the centre point of the tunnel section, and *R* denotes the distance between (*x_c_*, *z_c_*) to the fitting ellipse surface (Figure 4). The interference objects on the surface of tunnel linings with depth values less than 0 are set to 0 and the depth values of the anomaly points are set to 1. 

**Figure 5:**
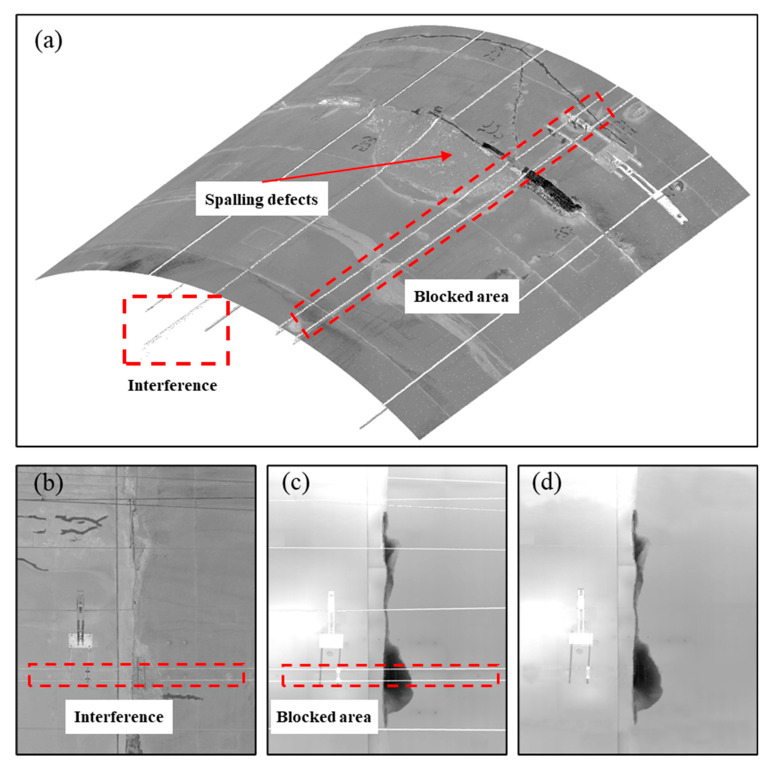
Removing the effect of interference: (**a**) tunnel point cloud data; (**b**) intensity image; (**c**) original depth image with interference parts, and (**d**) processed depth image without interference parts.

To enhance the texture of depth images, the power-law transformation is adopted in this paper [62]. As a result, the final grey value of the depth images can be computed by Equation (3):

(3) $$G_{d}={\left(1-D\right)}^{4}\times 255,$$

where *G_d_* denotes the final grey value in the depth images. Using this grey value conversion, the spalling region in the depth image moves towards black, and the lining surface has a mid-range grey value (Figure 5). As a result, the concrete spalling defects can be easily distinguished from the depth images. Parts of the concrete spalling region may be blocked by interference (e.g., utility cables, lights, monitoring instruments) on the tunnel lining surface (Figure 5b). Based on Equations (2) and (3), the depth values of the interference regions are all set to 0, and the corresponding grey values are computed as 255, and thus take on a white appearance in the depth image (Figure 5c). However, the white coloured region may bring obstacles to the boundary recognition and depth value quantification of spalling defects. As a result, the area and volume value calculation of the spalling defects may be inaccurate. To solve this issue, linear interpolation is used to estimate the depth value of the spalling region beneath the interference objects (Figure 5d), and the corresponding grey values of those regions shall be assigned by interpolating the adjacent non-interference pixels of the depth image.

### 2.3. Intensity and Depth Dataset for Spalling Detection

**Figure 6:**
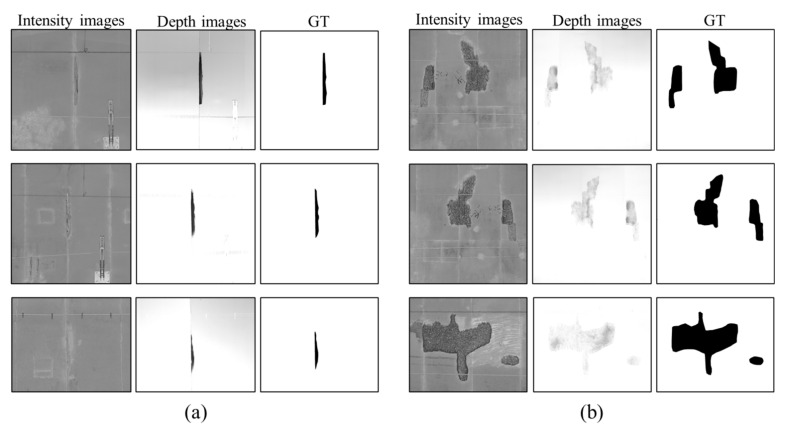
Some examples of the RTSD dataset, including: (**a**) spalling defects images with a large depth and (**b**) spalling defects images with a shallow depth.

This section establishes and introduces a dataset for the spalling segmentation task, the Railway Tunnel Spalling Defects (RTSD) with intensity and depth information. To improve the image processing efficiency, each original intensity image or depth image that contains a spalling region is cropped to a smaller size. During the cropping procedure, the coordinates of the four vertexes of the cropped depth image are recorded and used to crop the intensity images. These steps are processed by an algorithm to ensure the same position and size cropping is used for both types of images. Subsequently, the ground truth (GT) image of the spalling is then annotated manually by the annotation tool named LabelMe [63]. The labelling process is based on both the depth image and the intensity image information. The original RSTD dataset includes 1156 depth images and 1156 intensity images of the spalling region. Owing to the limited number of spalling images in this research, horizontal flipping, vertical flipping, random cropping, elastic twisting, local amplification, and brightness transformation are implemented to enlarge the dataset of spalling defects. The completed dataset contains 8092 intensity images, 8092 depth images, and 8092 GT images with respect to the same spalling defects (Figure 6).

The grey value of the depth images represents the depth value of concrete spalling in the actual environment, while the intensity image contains no quantitative depth information of the spalling defects (Figure 6a). For a concrete spalling defect with a shallow depth, a maintenance measure with epoxy resin coating may better highlight the spalling region in the intensity image than in the depth image (Figure 6b). Therefore, both the intensity image and the depth image are important for the evaluation of the spalling defects. The most significant feature of the RTSD dataset is to provide the missing depth information of the spalling defects compared with a traditional dataset which consists of only intensity/RGB images. 

## 3. SIDNet for Spalling Inspection

**Figure 7:**
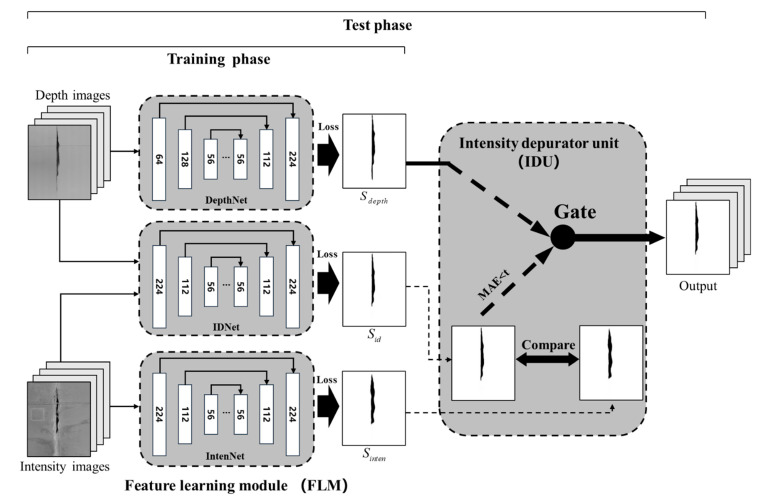
Framework illustration of the SIDNet model with the training phase and the test phase.

In accordance with the RTSD dataset, a DCNN-based model named SIDNet for automated segmentation and quantification of spalling defects is proposed in this study. It was developed based on the D^3^Net model [45], which consists of two major components: a feature learning module (FLM) and a depth depurator unit (DDU). The FLM model concludes three encode-decode modules for the semantic feature extraction. The DDU is used to remove the negative influence from low-quality depth images. For most samples in the RTSD dataset, the depth information contains more reliable spalling defects features than the intensity images. Therefore, in the proposed SIDNet, the original DDU in the D^3^Net model was replaced by a similar function module named the intensity depurator unit (IDU), which can adaptively filter the low-quality (Figure 6a) intensity images. In other words, the proposed SIDNet contains the FLM and the IDU modules. Figure 7 depicts the entire model, which can be divided into the training phase and the test phase. The IDU is only used in the test phase.

### 3.1. Three-Stream Feature Learning Module

**Figure 8:**
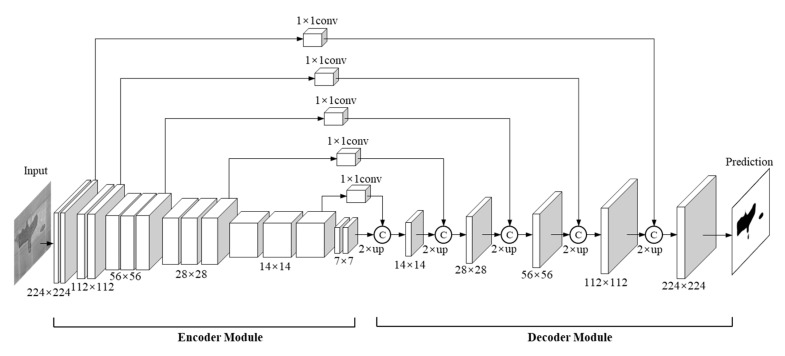
The structure of the three DCNNs (IntenNet, IDNet, and DepthNet).

The FLM consists of three parallel DCNNs named IntenNet, IDNet, and DepthNet, which have the same structure but a different input channel number. As shown in Figure 8, each DCNN of the FLM contains the input, encoder module, decoder module, and output. These three subnetworks receive and process three different input images (*I_inten_*, *I_id,_ I_depth_*) each with a resolution of 224 × 224 rescale pixels (i.e., the number of input channels is 1, 2 and 1). As a result, three independent prediction images (*S_inten_*, *S_id,_ S_depth_*) are outputted from these three subnetworks. The two-channel input *I_id_* consists of two types of images (intensity images and depth images) and the *S_id_* contains only normal one-channel output images.

During the training and testing phase, the DCNNs are used to extract features at multiple scales through a bottom-up pathway. The encoder module is modified on the base of VGG16 through a bottom-up pathway, which includes five convolutional stages and four pooling layers. To extract a deeper semantic feature in a deeper layer, a sixth stage is added at the end of the VGG16 structure with two 3 × 3 convolution kernels. The resolution of output in the first five stages corresponds to the VGG16 network (i.e., 64 × 224 × 224, 128 × 112 × 112, 256 × 56 × 56, 512 × 28 × 28, and 512 × 14 × 14), and the resolution is 32 × 7 × 7 in the sixth stage.

After the six convolution stages, six feature maps are captured with multi-level semantic information. Inspired by the feature pyramid network (FPN) [64], a similar module is designed for a multi-level semantic information extraction in a pyramid manner. For a specific coarse feature map, nearest neighbour operation is employed to achieve 2× up-sampling. To re-use the higher-resolution feature maps for richer information, the up-sampled feature maps are subsequently concatenated with the shallower feature maps using a lateral connection. During the decoder process, multi-scale feature maps (32 × 14 × 14, 32 × 28 × 28, 32 × 56 × 56, 32 × 112 × 112, and 32 × 224 × 224) are generated from the top-down pathway and the lateral connection. As a result, the prediction maps are correspondingly outputted from the end of the subnetworks using a 3 × 3 convolution layer. For the training phase, the widely used cross-entropy loss function is adopted to evaluate the difference between the GT image and the prediction image:

(4) $$L=-\frac{1}{N}\sum_{i=1}^{N}\left(g_{i}\log(s_{i})+(1-g_{i})\log(1-s_{i})\right),$$

where *g_i_* and *s_i_* are the pixels of the GT images and the prediction images, respectively, and *N* represents the total number of pixels. For the test phase, three independent output prediction images are inputted into the subsequent IDU module to get an optimized result.

### 3.2. Intensity Depurator Unit 

Despite the supplementary information provided by the depth images, the open-source RGB-D datasets still struggle at outputting high precision segmentation results due to the low quality of the depth images. Therefore, after obtaining three independent output maps (*S_inten_*, *S_id_*, *S_depth_*) from the FLM, the original D^3^Net includes a DDU module to generate an optimal prediction image by setting a filter to remove the effect of the low-quality depth images. Similarly, the purpose of IDU is to filter the low-quality intensity images. To evaluate the quality of the intensity image, the IDU in the proposed SIDNet firstly assesses the similarity between *S_inten_* and *S_id_* by calculating the mean absolute error (MAE) metrics.

(5) $$MAE(S_{id},S_{inten})=\frac{1}{N}\left|S_{id}-S_{inten}\right|,$$

where *N* denotes the total number of pixels. In theory, if the quality of the intensity image is low, the output of *S_inten_* will be quite different from *S_id_* as the latter has considered the more reliable depth feature. As a result, *S_depth_* shall be selected as the final prediction; otherwise, *S_id_* shall be considered as the optimal output of the model. A fixed threshold value, *t_inten_*_,_ is used to determine the quality of the intensity images, and the prediction of the SIDNet model can be concluded as:

(6) $$P=\{\begin{array}{c} S_{id},MAE(S_{id},S_{inten})\leq t_{inten}\\ S_{depth},MAE(S_{id},S_{inten})>t_{inten} \end{array},$$

By using the IDU module, the network can adaptively select the optimal result as output. The optimal *t_inten_* value will be determined in Section 4.2. To better understand the difference between the IDU module and the DDU module, the main principle of the DDU is shown using following equation:

(7) $$P=\{\begin{array}{c} S_{id},MAE(S_{id},S_{depth})\leq t_{depth}\\ S_{inten},MAE(S_{id},S_{depth})>t_{depth} \end{array},$$

where the parameter *t_depth_* is an evaluation index to the quality of the depth images, which filters the depth image information. For the established RTSD, most depth images contain more reliable spalling defects features than the intensity images. In this study, it is more important to filter the low-quality intensity images rather than the depth images. Hence, the proposed IDU module is more suitable for the spalling defects segmentation task. The performance comparison between the IDU module and the DDU module shall be discussed in Section 4.2.

### 3.3. Network Evaluation Metric

**Figure 9:**
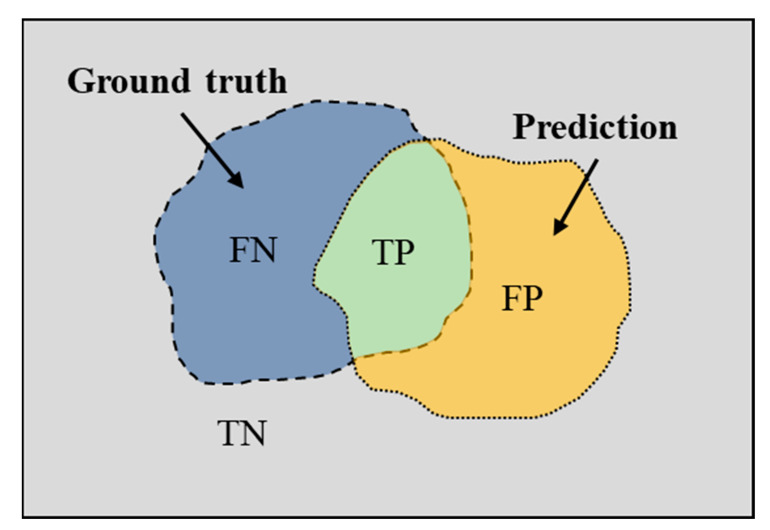
Relational diagram of the TP, TN, FP, and FN indicators.

For the semantic segmentation task, mean pixel accuracy (MPA) and mean intersection over union (MIoU) are the common metrics for evaluating the performance of the trained model. To calculate these two pixel-level segmentation metrics, four important parameters are firstly introduced: true negative (TN), true positive (TP), false negative (FN), and false positive (FP). Among these, TP and FP indicate the number of correctly segmented and falsely segmented pixels, and TN and FN denote the correctly unsegmented and falsely unsegmented pixel numbers, respectively. The correlation between TN, TP, FN, and FP is shown in Figure 9. In order to show the relationship between evaluation metrics and these four parameters, the calculation formula is shown as follows:

(8) $$MPA=\frac{1}{2}(\frac{TP}{TP+FP}+\frac{FN}{TN+FN}),$$

(9) $$MIoU=\frac{1}{2}(\frac{TP}{TP+FP+FN}+\frac{TN}{TN+FN+FP}).$$

### 3.4. Quantitative Evaluation of Spalling Defects

After segmentation via the SIDNet model, it can be determined whether the pixels in the images belong to spalling defects. The depth value between each spalling defect point and the lining surface can be calculated by reversing the depth projection process in Equation (3). As a result, the area of the spalling defects can be also obtained by counting the detected pixels from the segmentation results using the following equation:

(10) $$A=nh^{2},$$

where *h*^2^ denotes the actual area for each pixel (25 mm^2^ in this study), and *n* represents the number of pixels in this defect. Subsequently, the volume of the spalling defect can be computed by the following equation:

(11) $$V=\sum_{spalling}D_{i}h^{2},$$

where *D_i_* denotes the depth value of pixel *i* in the defects. 

## 4. Experiment and Results

Before the training process, the RTSD dataset was first randomly divided into three parts (training, validation, and test) with relative proportions of 70%, 20%, and 10%, respectively. As a summary, the image distributions in the RTSD dataset are listed in Table 1. The training and validation dataset was put into the model training phase for tuning the model parameters and avoiding overfitting. The testing dataset was used in the test phase to evaluate the segmentation robustness and accuracy of the trained model. In this study, the training and testing were carried out on a self-assembled desktop PC with an Intel Core i7-9700k CPU and an Nvidia GTX 2080Ti GPU with 12 GB of memory. The software environment was set with PyTorch framework on the Ubuntu 18.04 operating system.

### 4.1. Training and Test Results 

**Figure 10:**
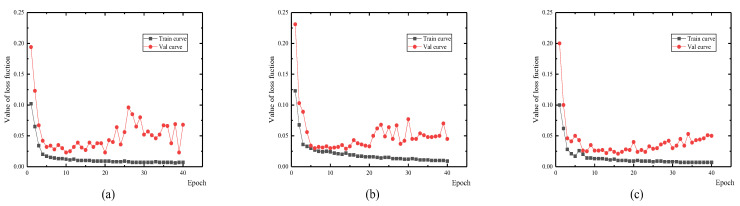
The learning curve of the subnetworks (**a**) IntenNet, (**b**) IDNet, and (**c**) DepthNet.

Three DCNNs were trained using 5664 depth images and 5664 intensity images. There were 1619 images used in the validation process. Among the three DCNNs, the hyper-parameters were set as the same with a batch size of 8, total epoch number of 40, and a learning rate of 10^4^ in the first 20 epochs, and 10^5^ in the remaining 20 epochs. During the training and validation phases, both the training and validation loss function curves of the three subnetworks showed a downward trend by updating the network weight. After 10 epochs, the validation loss values illustrated a rising trend, which suggests the networks were overfitting (Figure 10). To obtain the optimal performance, the network saved in the 10th epoch was outputted for the detection and evaluation of the spalling defects.

### 4.2. Segmentation Performance of the SIDNet

**Figure 11:**
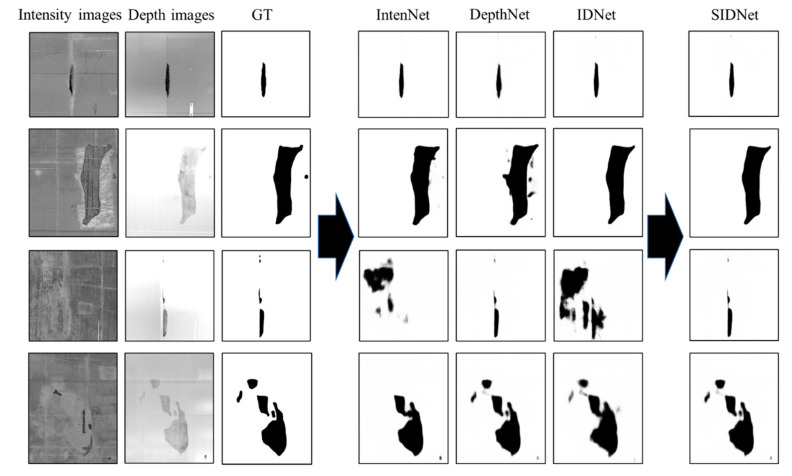
Examples of the spalling detection results output from the three DCNNs and the SIDNet.

**Figure 12:**
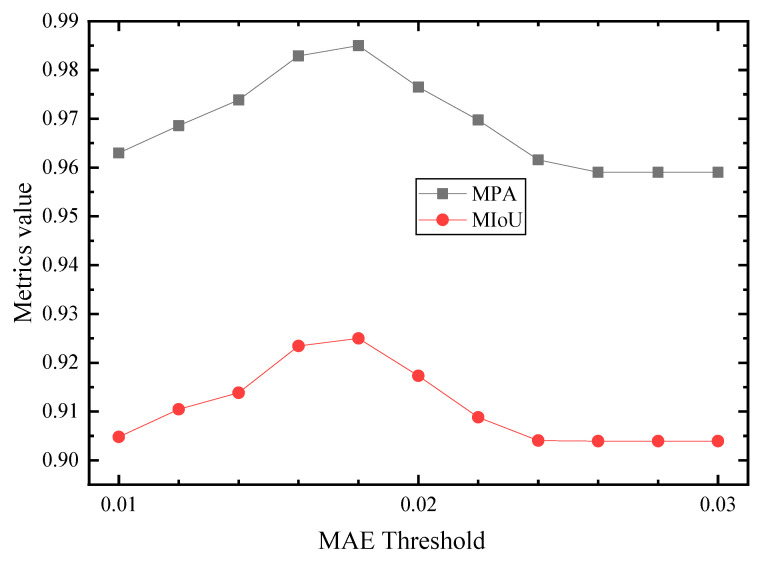
Experiment results of testing different MAE threshold values to obtain the optimal trained model.

The test dataset contains 809 intensity and depth images. Three output feature maps are generated from the three DCNNs. Subsequently, the optimal prediction will be determined by comparing the similarity (MAE value) of *S_inten_* and *S_id_* with the threshold *t_inten_*. With the IDU module, the high-quality intensity features (MAE value greater than *t_inten_*) retained (Figure 11, row 1 and 2), and low-quality intensity features (MAE value less than *t_inten_*) filtered out (Figure 11, row 3 and 4). The value of *t_inten_* thus affects the selection of the final output image. A set of *t_inten_* values (11 uniformly distributed values between 0.01 and 0.03) are further tested in this paper, and the model performance of each threshold is evaluated. Figure 12 shows that when *t_inten_* is 0.018, both the MPA and MIoU values of the model reach a maximum (0.985 and 0.925), which indicates that the model achieves the best performance. 

To verify the effectiveness of the IDU module, the performances of three DCNNs (IntenNet, IDNet, and DepthNet) were compared with the performance of the SIDNet and the D^3^Net. As shown in Table 2, the IDNet (0.970 and 0.911) performed slightly better than the DepthNet (0.957 and 0.905) in MPA and MIoU, and much better than the IntenNet (0.904 and 0.838). The D^3^Net with the DDU module shows a better performance than the IntenNet but a lower performance than the IDNet and the DepthNet. The relative low performance of the D^3^Net is caused by filtering the depth feature. Therefore, the depth feature is more reliable than the intensity information in the semantic task of segmenting tunnel spalling defects. Meanwhile, the SIDNet model with the IDU module shows the best performance compared to the three DCNNs. The IDU’s ability to discard the low-quality intensity feature and choose an optimal path (*S_id_* or *S_depth_*) contributes to its superior performance. The metrics of SIDNet and D^3^Net also prove the IDU’s superiority to the DDU.

To examine the segmentation results of the proposed method, the traditional Otsu segmentation algorithm is used in this paper for comparison. The performance of the SIDNet is also compared with two widely accepted DCNN-based segmentation algorithms: DeepLabV3+ and UC-Net. These two semantic segmentation models are end-to-end frameworks that can perform high precision semantic segmentation at the pixel level. For consistency, the training dataset and basic training parameters of DeepLabV3+ and UC-Net are the same as those of the RTSD model. UC-Net is a state-of-the-art semantic segmentation algorithm considering both intensity and depth information of the target feature, and DeepLabV3+ is a classic DCNNs model based on only the intensity information of the images. Hence, the depth information is not used in the DeepLabV3+ and Otsu models. The metric results of the three models are also listed in Table 2. The MPA and MIoU values of the proposed method (0.985 and 0.925) were slightly higher than those of UC-Net (0.971 and 0.907). With the benefit of the extra depth information, the proposed SIDNet model showed much higher values in MPA and MIoU than the DeepLabV3+ (0.881 and 0.792) and Otsu (0.519 and 0.409) algorithms. 

**Figure 13:**
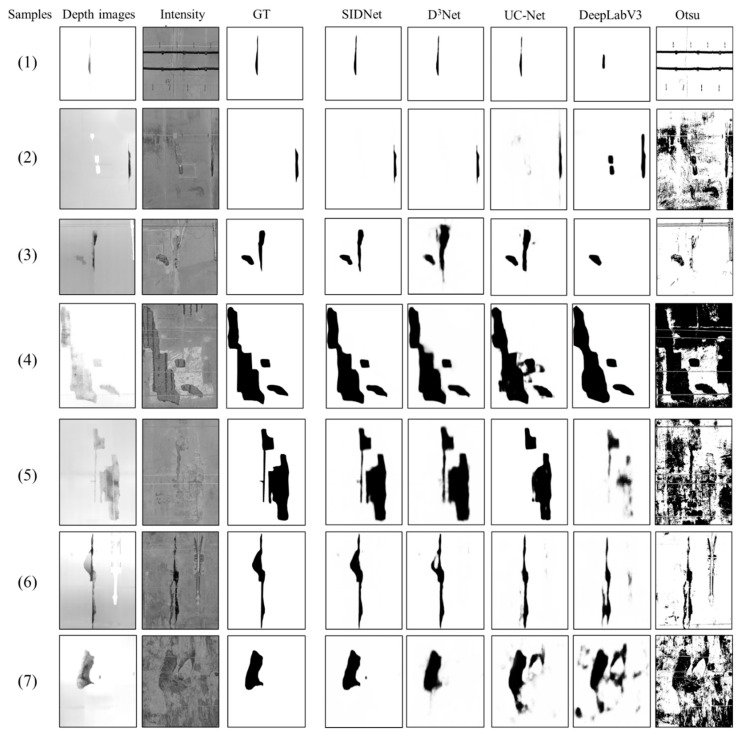
Comparison of the inspection results using several different algorithms, including deep learning-based methods and the traditional threshold segmentation method.

By selecting seven testing samples, the proposed SIDNet model showed a much more effective performance than DeepLabV3+ (Figure 13), and the Otsu algorithm achieved the worst performance at recognising spalling defects and noise reduction. Compared to D^3^Net and UC-Net, the IDU module can help the proposed SIDNet model to explicitly eliminate the effect of low-quality intensity images and extract only the most effective depth information (Figure 13, sample 4 to sample 7). 

### 4.3. Evaluation of the Detected Spalling

**Figure 14:**
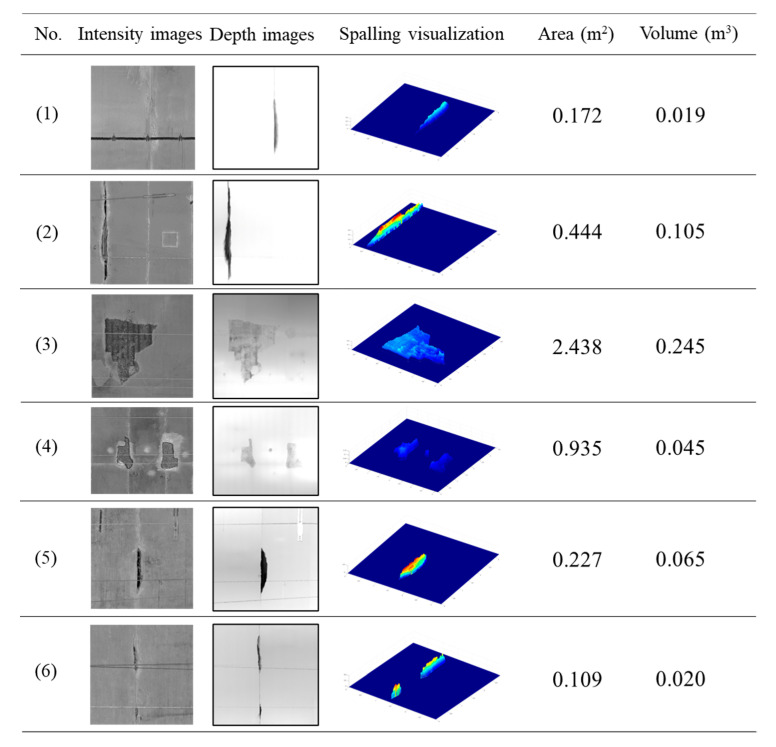
Detection and quantification results of six test samples.

As mentioned in Section 3.4, the spalling quantification information (area and volume) can be obtained by counting the predicted pixels and summing the actual depth values belonging to the spalling region. Using six test samples as an example, Figure 14 shows the detection and the statistical quantification results of the detected defects. The input images (intensity and depth images) are presented, and the spatial shape of the spalling defect is plotted using a colour distinction (blue to red) to represent the actual depth value (0 to 0.5 m). The computed area and the volume value are also given. 

**Figure 15:**
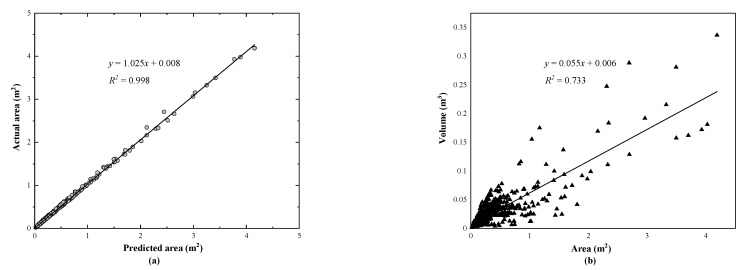
Comparison evaluation of the prediction indexes, including: (**a**) predicted and actual areas of the spalling defects and (**b**) predicted area and predicted volume.

To further explore the relationship between the area and volume values of the spalling defects, the predicted and the actual spalling (GT region) areas of all the test images are plotted in Figure 15a. The statistical results suggest that the gradient and the *R*^2^ of the linear regression line are equal to 1.025 and 0.998, respectively, which indicates a high accuracy of the proposed model. Similarly, Figure 15b demonstrates the statistical result of the actual area and the volume of the spalling defects. Since the ground truth volume values of the spalling defects are not available, the computed volume can be regarded as the reference. There is a weak linear relationship between the values of area and volume (*R*^2^ of 0.733). 

The results in Figure 15b suggest that, in most cases, a larger defect area means a larger volume, but in a considerable number of cases, if the actual spalling depth value is too large or too small, the opposite conclusion should be reached. Although the acquisition of depth images has become more convenient with the increasing application of depth sensors (such as Microsoft Kinect and smart phones), the relative low precision of the obtained depth value is still an unsolved issue. Traditionally, the photogrammetry-based inspection methods mainly focus on the 2D evaluation information (segmentation result and area value) of the defects. The missing of high precision depth value may lead to the tunnel inspectors overestimating a spalling defect when it has a large area but shallow depth. In fact, the volume reflects the quantitative index of structural damage caused by the spalling defects. The deep depth of spalling often means that the steel in the precast reinforced concrete lining is exposed to the environment, making it more susceptible to corrosion (Figure 13, row 3). At the same time, the obtained volume value can provide reference for the required amount of materials needed for tunnel lining maintenance, which is in turn beneficial for structure health monitoring and maintenance of an operational railway tunnel. 

### 4.4. Robustness Test on the Proposed Approach

**Figure 16:**
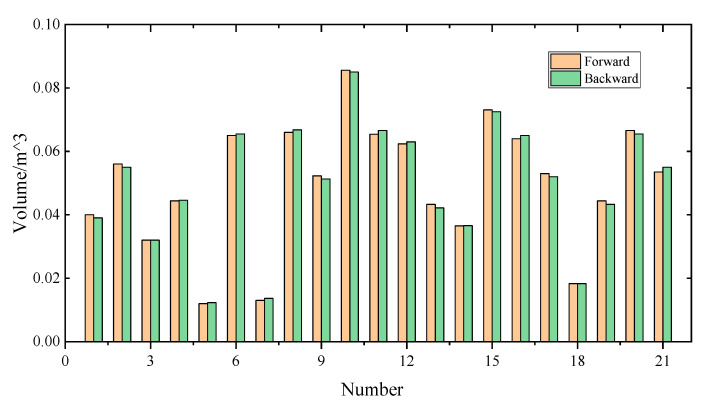
Comparison results of volume calculation from forward and backward measurements.

To evaluate the robustness of the proposed approach, two sets of point cloud data were obtained separately via forward and backward measurements along a 500-metre-long tunnel region. This region contains 21 spalling defects. The root mean square error (RMSE) is adopted to compare the difference of the depth value from two groups of spalling point cloud data, which can be defined as the following:

(12) $$RSME=\sqrt{\frac{\sum_{i=1}^{n}\Delta_{i}}{n}},$$

where *n* is the number of pixels belonging to spalling region, and ∆ is the difference in the actual depth values. The RMSE in this paper is 0.9 mm, which is close to the precision of the laser scanner in theory (0.5 mm@10 m). The relatively low RMSE value reflects a high internal precision of the MLS system and reliability of the calculated volume of the inspected spalling defects. The volume values of 21 spalling defects for both datasets are plotted in Figure 16. The statistical results show that the mean error is 1.66% and the max error is 4.85%, which suggests a high robustness of the proposed approach. 

## 5. 3D Visualization and Inspection Report 

### 5.1. 3D Tunnel Model Reconstruction Method

To visualize tunnel structure defects in 3D space, the predicted 2D images must be visualized in 3D space to allow the inspectors to better assess the spalling defects in the tunnel linings. An effective solution is 3D tunnel reconstruction based on the obtained images and adding topological information to the original point cloud data. 

**Figure 17:**
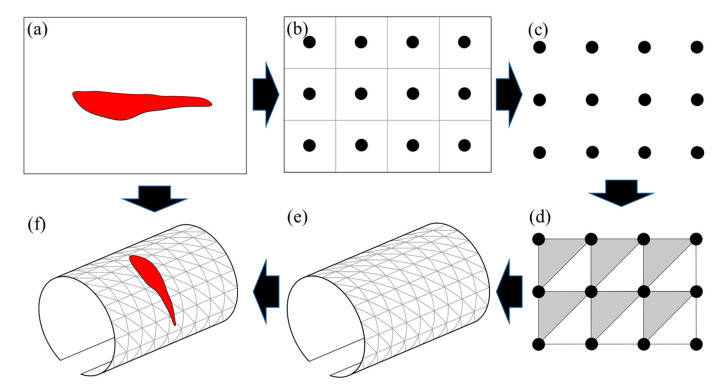
Workflow of the 3D tunnel model reconstruction, including: (**a**) the predicted defect inspection image from the SIDNet model; (**b**) generating 2D points; (**c**) generating 2D plane point cloud; (**d**) forming a triangular mesh surface; (**e**) 3D tunnel lining surface reconstruction; (**f**) mapping defect inspection images on the surface of tunnel model.

Figure 17 shows the main steps of the tunnel 3D reconstruction approach introduced in our previous work [55]. Based on the predicted images from the SIDNet model, a custom 2D plane point cloud is first generated by placing a point at the centre of the corresponding position pixel (Figure 17a–c). To convert from the 2D point cloud to a 3D surface model, a diagonal triangulation mesh method is used to generate the surface mesh. Based on the plane point cloud, three nearest neighbouring points are connected to form a triangular mesh surface (Figure 17d). Through batch processing, all the point cloud data can be efficiently transformed into a mesh surface of the same size as the images. In order to reconstruct the 3D tunnel lining surface, the obtained 2D mesh surface should be transformed into the spatial space. To simplify the reconstruction process and increase the processing efficiency, the 2D points (including the points on the interference objects) are mapped on the 3D surface of a fitted ellipsoid tunnel lining (Figure 17e). The assumed 3D ellipse tunnel model has the same coordinates as the fitted ellipsoid tunnel introduced in Section 2.2. The spalling defect inspection images output from the SIDNet are then mapped on the surface of the constructed 3D model according to the projection relationship between 2D pixels and 3D points. As a result, the final 3D tunnel lining model consists of both spatial location information and colour information (Figure 17f). 

### 5.2. 3D Inspection Results of a Testing Tunnel Section

A 75 m long tunnel section was selected to demonstrate the capability of the proposed approach. The 3D point cloud data of each 5 m length were first converted into intensity images and depth images. These images were input into the trained SIDNet model and then output with the same size. After obtaining numerous individual images with segmented spalling defects from the SIDNet model, the relatively small output images were stitched together directly to form a larger one, aiding successive inspection and reducing the number of images. 

**Figure 18:**
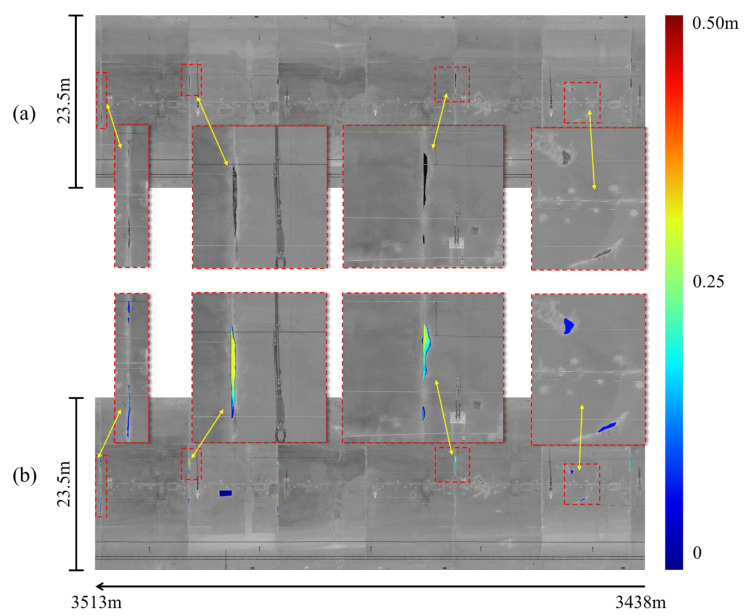
The comparison from a 75 m long tunnel section between (**a**) the stitched input intensity image and the enlarged local regions and (**b**) the inspection results output from the SIDNet model and the enlarged spalling defects regions (the depth value ranges from 0 (blue) to 0.5 m (red)).

The relationship between the relative positions of the spalling defects can be accessed in the context of the final mosaic image. Figure 18a is the stitched input intensity image of the 75 m tunnel region and the actual length of image’s width is 23.5 m. The corresponding inspection results output from the SIDNet model are shown in Figure 18b. The inspected spalling defects with different depths (ranging from 0 to 0.5 m) are marked with different colours (blue to red) to demonstrate the quantification results of the detected defects in the images. Overall, the spalling defects in this tunnel region are successfully segmented and quantified. 

**Figure 19:**
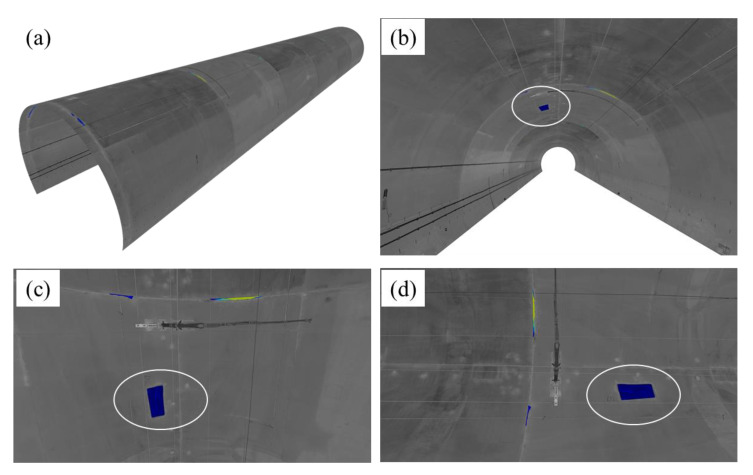
3D visualization results from several viewing perspective of the detected spalling defects including: (**a**) overall view, (**b**) longitudinal view, and (**c**,**d**) front view; a specific spalling defect is highlighted with white circles from different viewing perspective.

A 3D model of the 75 m tunnel region was constructed based on the predicted image which shows different viewing perspectives of the tunnel lining (Figure 19). The 3D model also provides an intuitive 3D view of the location and distribution of the detected spalling defects. By using depth images, the depth of spalling can be visualized in the form of different colour distinctions. If needed, the interference objects can be also eliminated in the reconstructed 3D model. A spalling inspection report can also be automatically generated (Table 3). In this report, the longitudinal location (the mileage according to the tunnel entrance that can be seen in the Figure 18), the according angle range of each spalling defect (the start and end angle relative to the *x* axis, which has been introduced in Huang et al. [55]), the area, and the volume of the identified spalling defect are accurately quantified and outputted. This automated spalling inspection approach can significantly aid with tunnel structural health monitoring and improve the digital management of railway tunnels.

## 6. Discussion

### 6.1. The Advantages of the Proposed Approach

To estimate the required amount of material for the maintenance of spalling defects, both the area and volume of each spalling defect must be accurately quantified. During the traditional manual inspection of spalling defects, the spalling region is subjectively sketched or estimated by the inspector, and hence the defect area is roughly estimated. Total station and Vernier callipers have often been used to acquire the depth information of the spalling defects. However, both kits are difficult to operate in a railway tunnel and can only make single spot measurements. Therefore, the calculated volume of manually detected spalling defects is not accurate. 

In recent years, photogrammetry has developed rapidly as a non-destructive inspection method for tunnel structure health monitoring [33,34,35,65]. By mounting high-resolution linear charge coupled device (CCD) cameras onto a movable inspection platform, photogrammetry can achieve continuous scanning imaging of the tunnel lining surface along the longitudinal direction. The obtained high-resolution images are used for the detection and quantification of defects on the tunnel lining (water leakage, cracks, and spalling). However, only the 2D evaluation information (segmentation result and area value) of the defects can be obtained. The depth information and 3D spatial coordinates of the spalling defects are hard to calculate. Therefore, it is difficult to obtain the volume of the detected spalling defects. 

The integrated approach proposed in this paper can calculate the actual depth of spalling defects through the acquisition of high-precision 3D coordinate values. The depth channel combining the intensity channel can be imported into the DCNNs to improve the semantic segmentation accuracy of the model, so that the volume of the spatial spalling defects can be calculated precisely. The point cloud data of 3D tunnel linings can be collected from the MLS with high efficiency and density, providing a means to capture the depth features in a small defects area, and the volume of spalling defects can be computed by means of accumulation. Moreover, the 3D defects visualization and automated inspection report generating approach can surely improve the efficiency and enable better documentation of the tunnel inspection operations.

### 6.2. Possible Applications of the Proposed Approach

It should be noted that the unrolling step of the proposed approach is restricted to the cases of circular or ellipsoid tunnel. In general, the railway or metro tunnel linings can fit well with an ellipse surface. Studies shall be conducted in future work to allow spalling defects inspection in roadway tunnels with different tunnel lining geometries. Nevertheless, the proposed approach in this study should be applicable for the detection of spalling defects in metro shield tunnel linings. The precision of the depth information from point cloud data will be improved as a metro shield tunnel has a smaller radius. At the same time, a smaller inner surface means that more point cloud data will be collected in a certain size spalling region, thus improving the density of depth value estimation. 

According to research [14,66], the extra depth information has proved to have positive effects in structural defect inspection and quantification. In theory, by employing the proposed approach, it should be able to segment and quantify all the target objects (bolt holes, segment joints, dislocations, etc.) using depth inconsistency. Adopting the depth feature can significantly advance tunnel lining inspections by overcoming the problem of interference objects having similar colours.

## 7. Conclusions

This paper proposes a novel integrated approach for automated segmentation and quantification of spalling defects from tunnel lining point cloud data. A dataset for spalling segmentation named RTSD is established from the point cloud data, comprising 8092 RGB images and depth images of railway tunnel linings. Owing to the additional depth information, a DCNN-based model named SIDNet is proposed to achieve multichannel feature extraction of spalling defects. The depth information helps to quantify the volume of spalling defects of the tunnel lining. The SIDNet model exceeds the performance of the other state-of-the-art DCNN-based and traditional models with regards to the MPA (0.985) and MIoU (0.925). The area and volume values can be quantified from the output images to evaluate the detected spalling defects. 

This study also integrates the 3D reconstruction method to better visualize and quantify the inspected defects in 3D space. A 3D surface model of a tunnel lining can be generated to create a first-person observation perspective to the detected defects. The visualization approach helps an inspector to contextualize the location of the spalling defects found during inspection in an intuitive manner. A 3D inspection report can also be generated automatically, to include the spatial location and the quantification values of the identified spalling defects. 

It should be noted that the precision of the depth value measurement and resolution of the point cloud are limited owing to the point cloud density. In future work, the authors will consider integrating high-resolution linear CCD cameras with the MLS to achieve higher accuracy in inspecting tunnel defects. Further advancement of the current spalling defects inspection practice should include testing the applicability and capability of the proposed approach in different types of railway tunnels and metro shield tunnels.

## Figures and Tables

**Table 1:** The images distribution of the RTSD dataset.

Category	Training	Validation	Testing	Total Number
Intensity	5664	1619	809	8092
Depth	5664	1619	809	8092
GT	5664	1619	809	8092

**Table 2:** Statistical results of different algorithms for segmenting spalling defects.

	IntenNet	DepthNet	IDNet	SIDNet	D^3^Net	UC-Net	DeeplabV3+	OTSU
MPA	0.904	0.957	0.970	0.985	0.935	0.971	0.881	0.519
MIoU	0.838	0.905	0.911	0.925	0.874	0.907	0.792	0.409

**Table 3:** Inspection report for spalling defects from a 75 m long tunnel section.

Spalling No.	Mileage (m)	Start Angle (°)	End Angle (°)	Area (m^2^)	Volume (m^3^)
#1	3438	132	134	0.031	0.017
#2	3438	101	120	0.187	0.028
#3	3446	96	103	0.086	0.004
#4	3445	79	82	0.103	0.004
#5	3463	103	122	0.254	0.053
#6	3463	92	94	0.037	0.003
#7	3488	115	121	0.107	0.017
#8	3488	64	73	0.089	0.012
#9	3495	80	85	1.253	0.075
#10	3501	100	121	0.356	0.068
#11	3501	76	80	0.121	0.010
#12	3513	117	119	0.118	0.009
#13	3513	115	116	0.065	0.004
#14	3513	63	85	0.347	0.031

## Data Availability

Not applicable.
